# Supplementary material for: Keypoint-based modeling reveals fine-grained body pose tuning in superior temporal sulcus neurons
Source: Nat Commun. 2025 Jul 1;16:5796. doi: 10.1038/s41467-025-60945-5 (PMC12214754; doi:10.1038/s41467-025-60945-5)
Supplement: Supplementary file 1 — Supplementary Information [file 41467_2025_60945_MOESM1_ESM.pdf]

Supplementary Information

Keypoint-Based Modeling Reveals Fine-Grained Body Pose Tuning in Superior Temporal Sulcus Neurons

Supplementary Figures (20), Table (1), and Supplementary text (related to Figure S9).

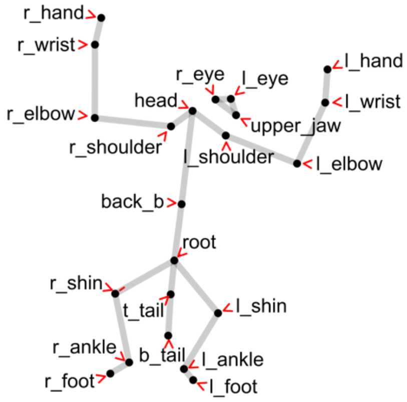

Figure S1. Keypoints of the avatar. *The 22 keypoints are indicated on a stick figure of a monkey (l = left; r = right).*

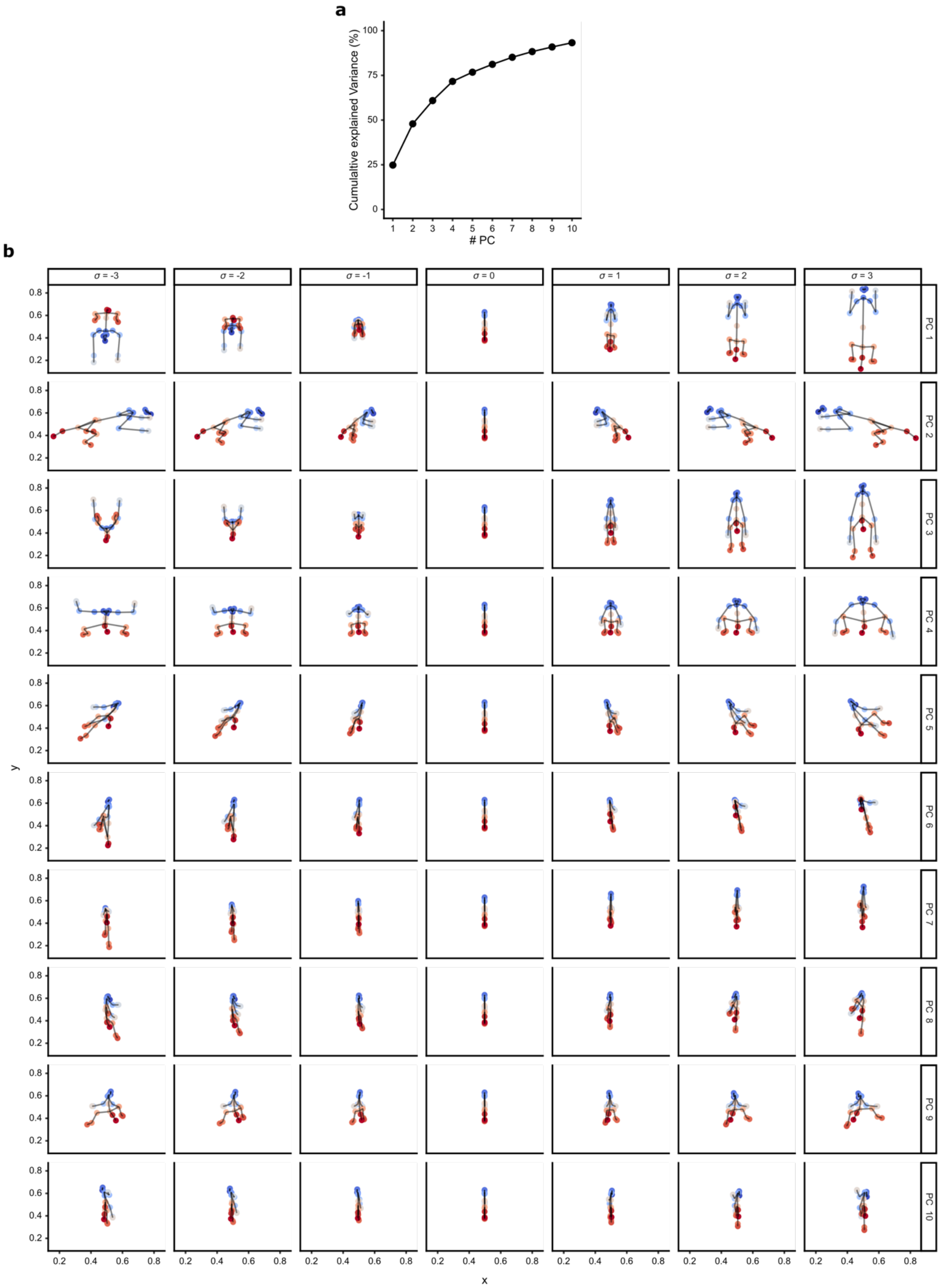

Figure S2. Principal component analysis of 2D keypoints. ***a***, *Cumulative explained variance plotted against the principal components.* ***b***, *Eigenposes associated with each principal component, displayed for standard deviations ranging from [σ = -3 to σ = 3] along the axis. Each keypoint is plotted with a different color.*

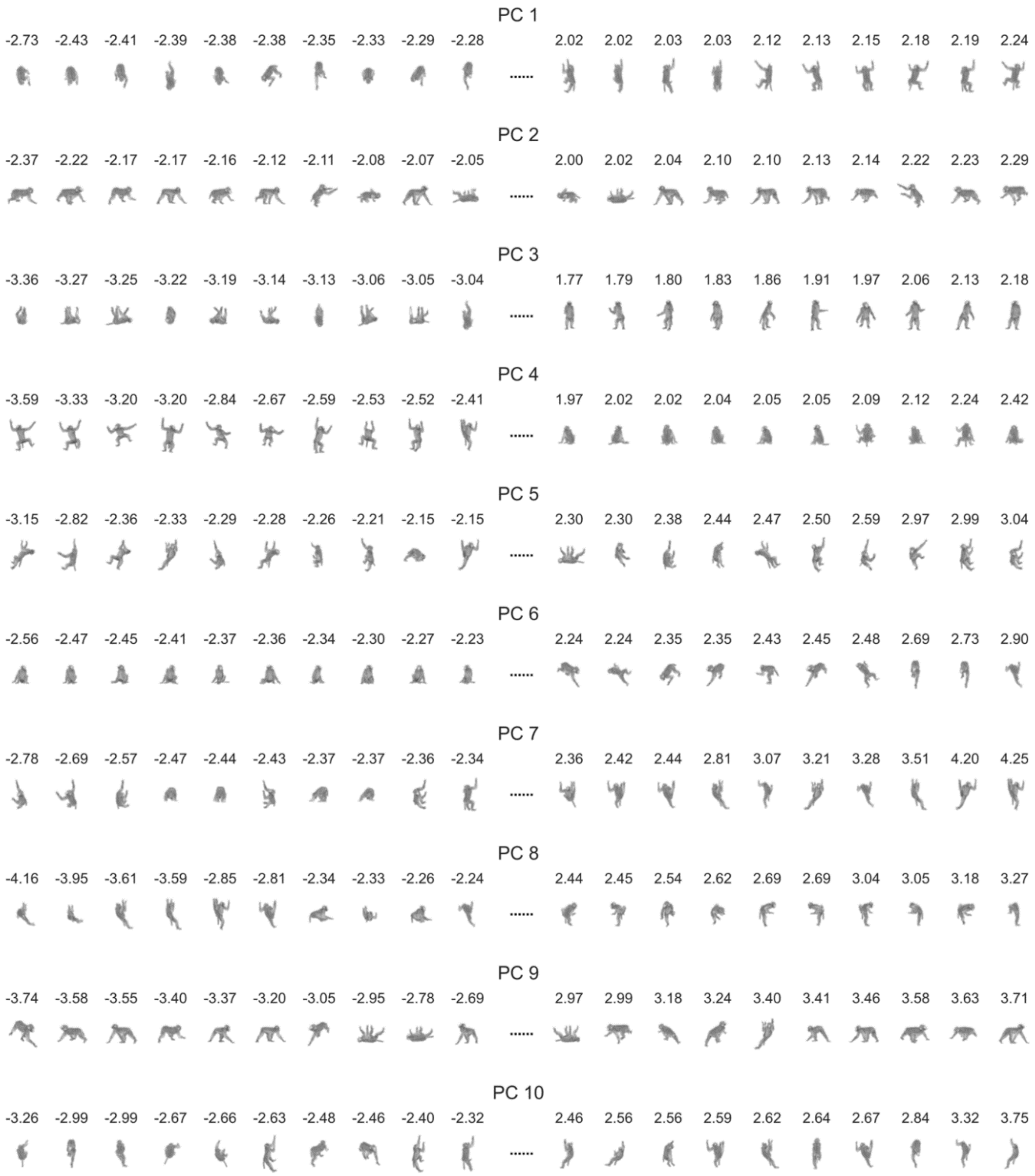

Figure S3. Stimuli along the principal components for the 2D keypoints. *Stimuli were ranked based on their standardized scores along the 10 principal component axes. The 10 stimuli with the most positive (right) and most negative (left) standardized scores for each PC are displayed, along with their corresponding score values**.** As expected there is some correspondence between the eigenposes of Figure S2 and the top 10 or bottom 10 images for those PCs, e.g. the change in viewpoint for PC2 is obvious for the top versus bottom images of that PC. However, images with high scores for a particular PC can be quite different, e.g. the different views for the top images for PC1. These differences are captured by other PCs.*

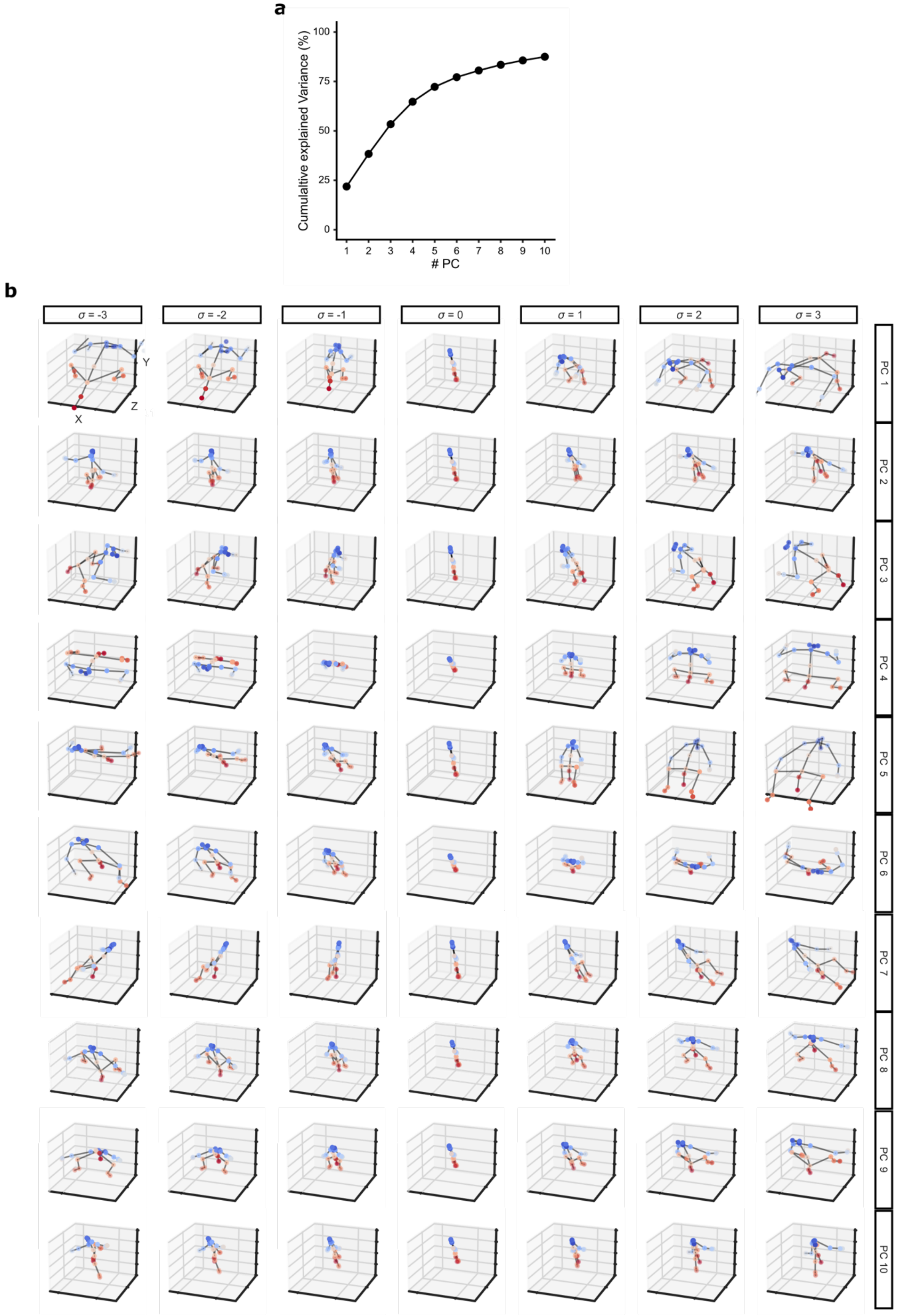

***Figure S4. Principal component analysis of 3D keypoints. a**, Cumulative explained variance plotted against the principal components. **b**, Eigenposes associated with each principal*

component, displayed for standard deviations ranging from [σ = -3 to σ = 3] along the axis in 3D. Keypoints are plotted with a different color.

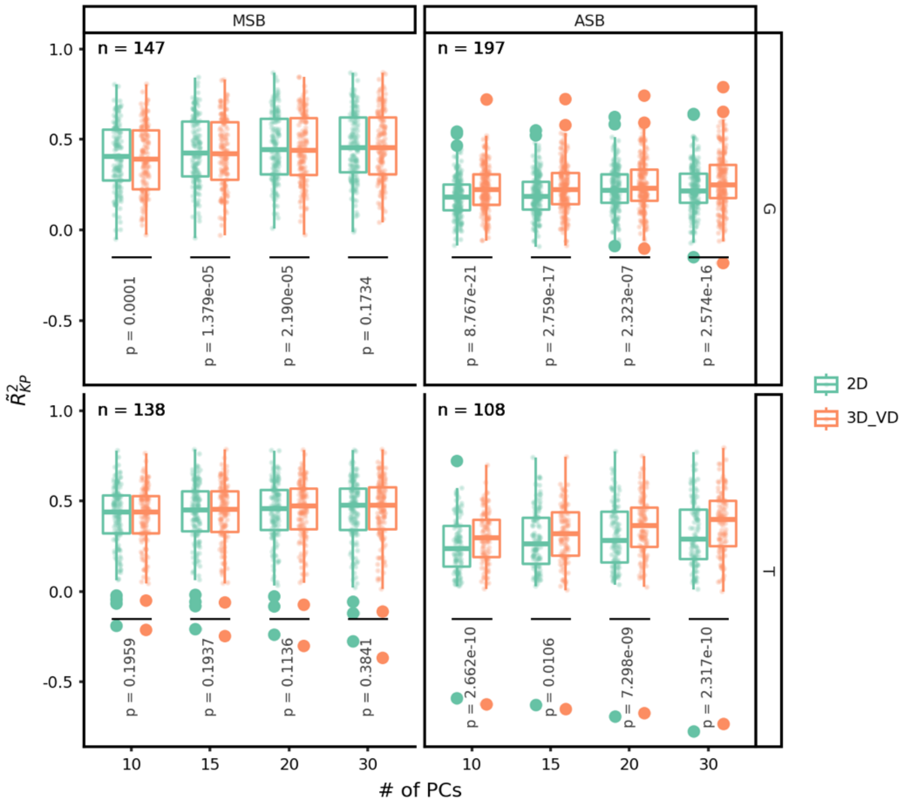

Figure S5. Box plots of reliability-normalized $\widetilde{R}^2$ for different numbers of selected PCs of the keypoint 2D and 3D_VD models. *The p values of are from two-sided Wilcoxon signed rank tests. Columns are for the regions while rows correspond to the monkeys. Box plots show the median (horizontal line), interquartile range (box: 25th–75th percentile), and data within 1.5× the interquartile range from the lower and upper quartiles (whiskers). Points beyond this range are plotted individually as outliers. N indicates the number of units.*

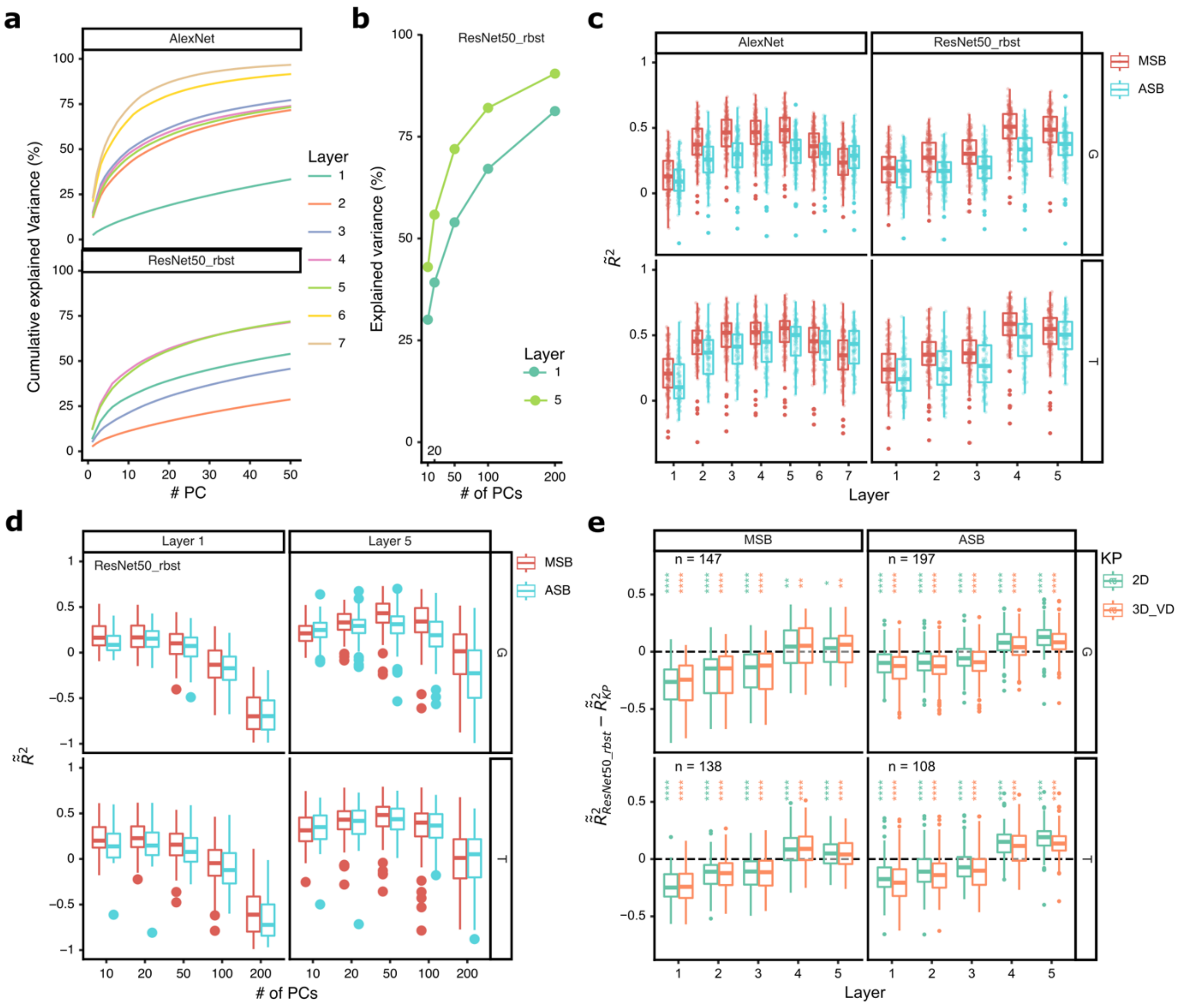

Figure S6. CNN modeling. ***a****, Cumulative explained variance plotted against the principal components for AlexNet (top) and for ResNet5_rbst (bottom) for each layer.* ***b,*** *Cumulative explained variance as a function of the number of selected principal components for layers 1 and 5 in ResNet50_rbst.* ***c****, Distribution of R^2 (without reliability normalization) of MSB and ASB units of both monkeys (row panels) for the model build for each layer of the networks (column panels).* ***d****, Distribution of reliability-normalized and adjusted R^2, depicted as $\tilde{\tilde{R}}$****,*** *as a function of the total number of selected PCs (see* ***b****) of layer 1 and layer 5 activations (column panels), for the units in MSB and ASB of both monkeys (row panels). For unadjusted R^2 data, see Figure S7.* ***e****, As in the main Figure 2b, the distribution of the difference of the reliability-normalized adjusted coefficient of determination between the keypoint-based model and ResNet50_rbst. For unadjusted R^2 data, see Figure S7. **** p < 0.0001; ***p<0.001; **p<0.01; *p<0.05; ns p >0.05; p values of two-sided Wilcoxon signed rank tests. Columns are for the regions while rows correspond to the monkeys. Box plot convention as in Figure S5. N indicates the number of units.*

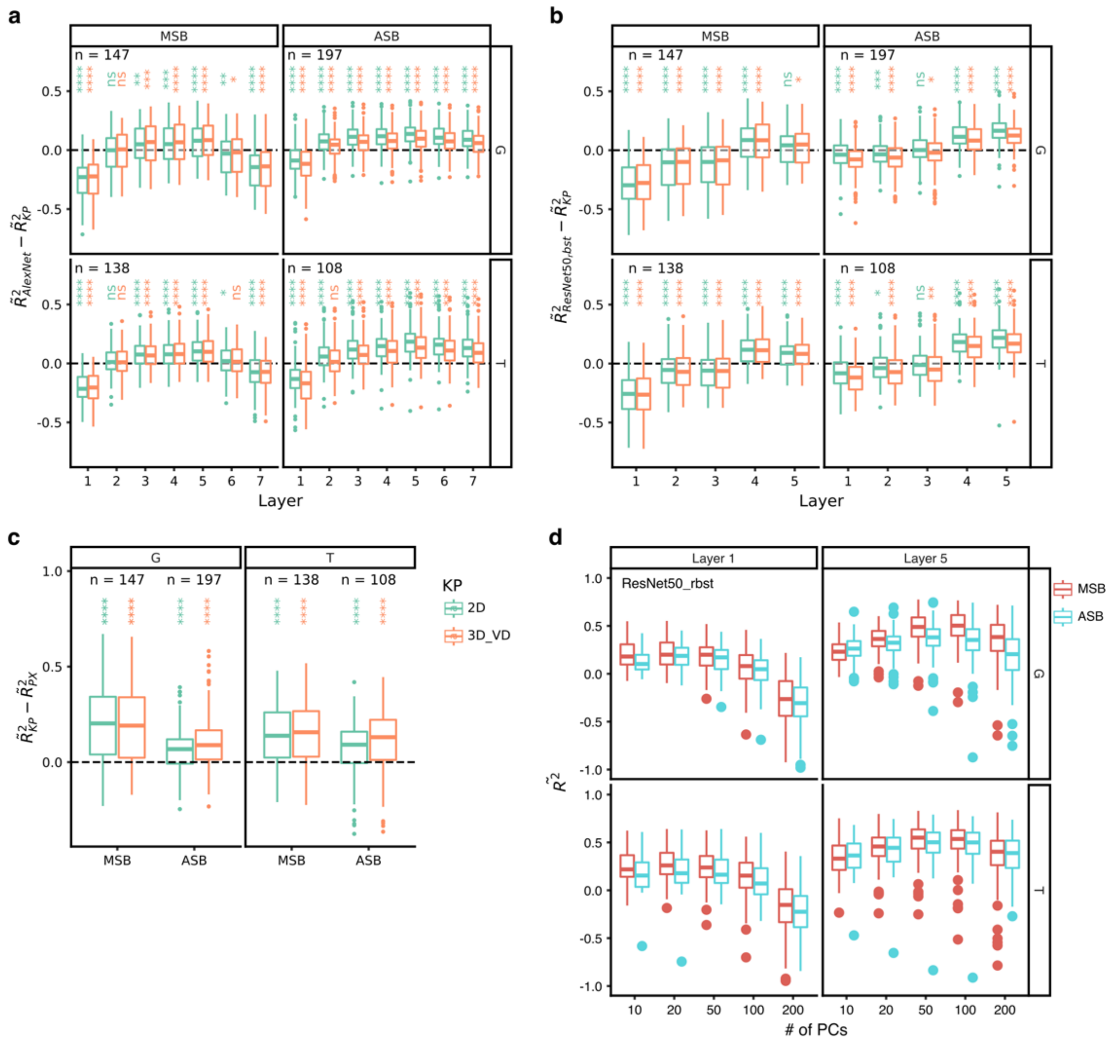

***Figure S7. Comparison of models using reliability-normalized but unadjusted $\widetilde{R}^2$. a**, Comparison between AlexNet and view-dependent keypoint models (2D (blue) and 3D_VD (red)); Same conventions as for Figure 2b of the main text.* ***b**, Comparison between ResNet50_rbst and view-dependent models (2D and 3D_VD). The same conventions as for Figure S6e.* ***c**, Comparison between pixel-based model (Figure S8) and view-dependent models (2D and 3D_VD). The same conventions as in Figure S8c.* ***d**, Distribution of $\widetilde{R}^2$ for ResNet50_rbst as a function of the total number of selected PCs. The same conventions as in Figure S6d. **** p < 0.0001; ***p<0.001; **p<0.01; *p<0.05; ns p>0.05; p values of two-sided Wilcoxon signed rank tests. Box plot convention as in Figure S5. N indicates the number of units.*

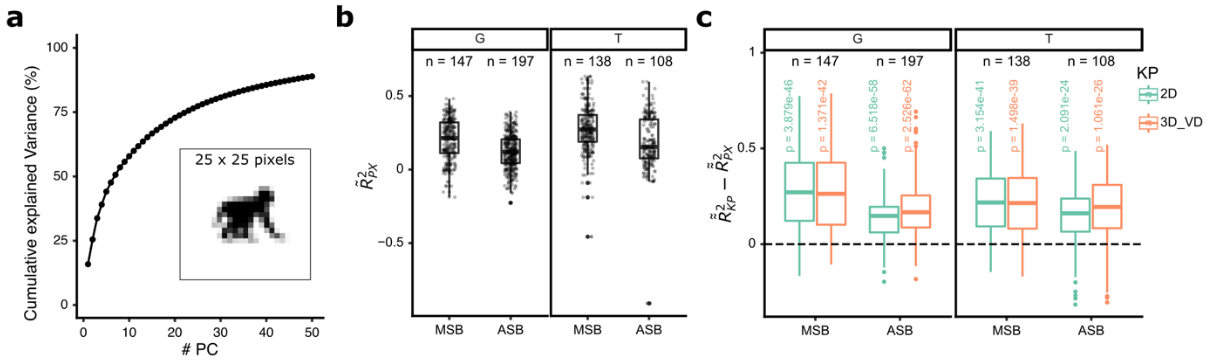

Figure S8. Pixel-based model analysis. a, *Cumulative explained variance plotted against the principal components for downsampled silhouettes of the poses, keeping the overall shape. Inset shows an example 25 × 25-pixel image used, along with 719 others, to obtain the principal components.* ***b****, Distribution of reliability-normalized coefficients of determination for each unit in the MSB and ASB regions of both monkeys (columns).* ***c****, Distribution of the difference between reliability-normalized adjusted coefficients of determination for the pixel-based model compared to the keypoint-based model. Unadjusted data are shown in Figure S7c; p-values from two-sided Wilcoxon signed-rank tests are shown for each region and monkey (columns). Similar results were obtained when downsampling to 10 x 10. Box plot convention as in Figure S5. N indicates the number of units.*

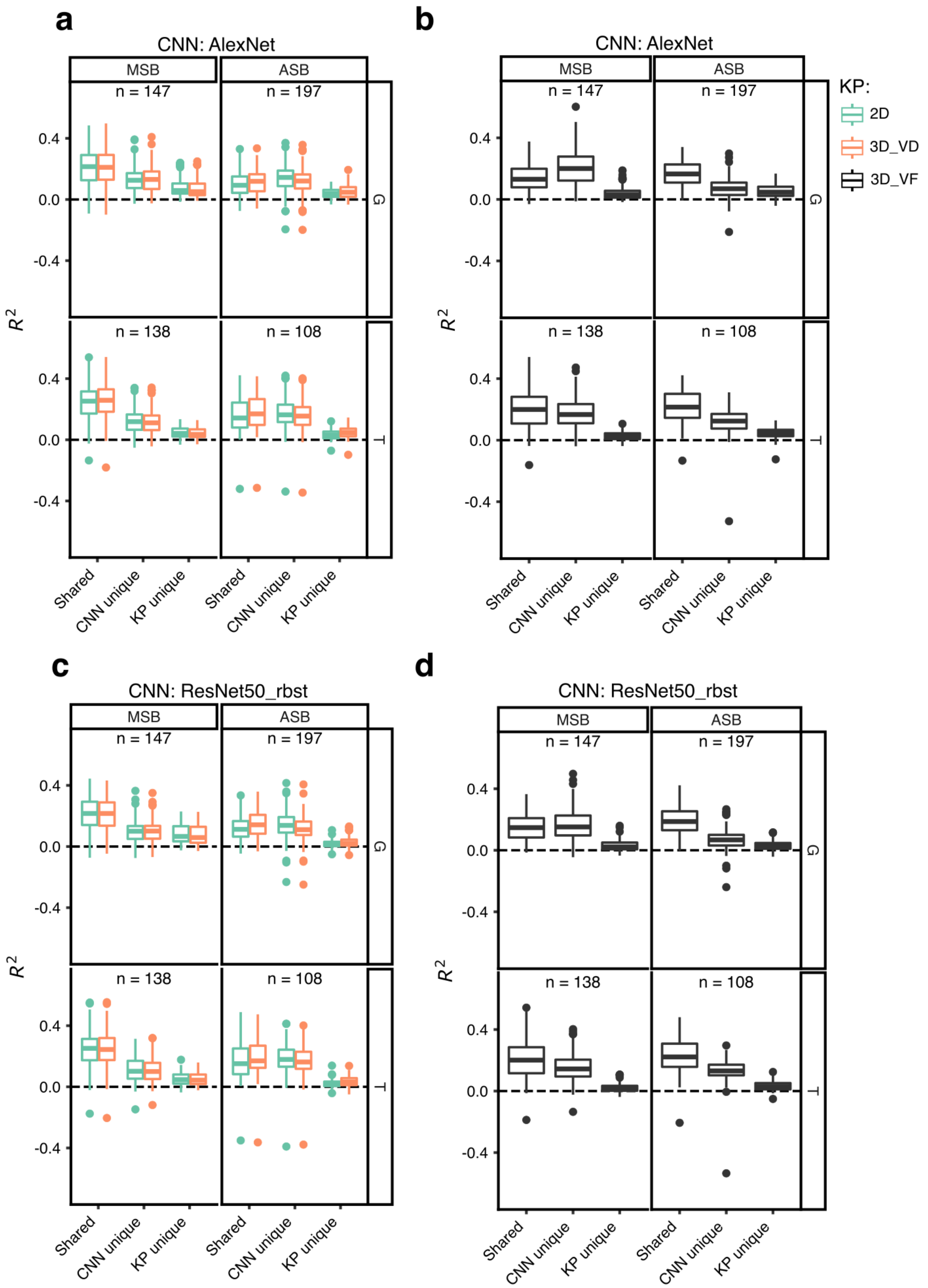

Figure S9. Variance partitioning analysis. *Box plots of* R^2 *according to the variance partition analysis for* ***a****, AlexNet and 2D and 3D_VD keypoint (KP) models,* ***b****, AlexNet and the 3D_VF keypoint model (see Figure S),* ***c****, ResNet50_rbst and 2D and 3D_VD models and* ***d****,*

*ResNet50_rbst and the 3D_VF model. See “*Supplementary text related to Figure S9*” for more details and discussion of the results.*

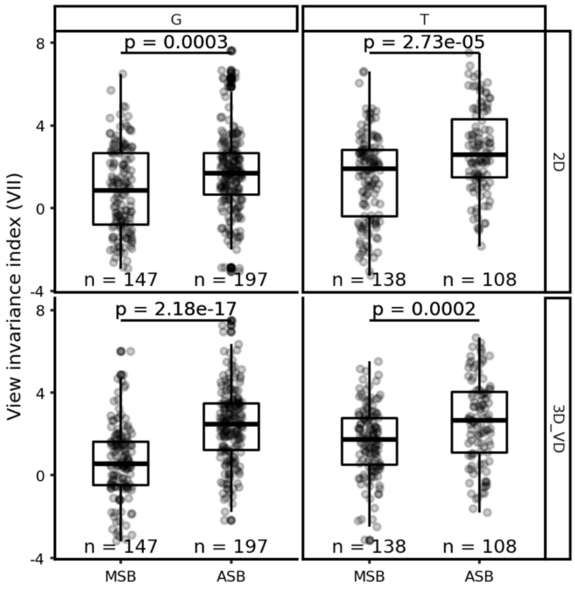

Figure S10. Distribution of the View Invariance Index (VII) for all the units. *The difference between the median VII for MSB and ASB was significant in each region and monkey (p values for two-sided Wilcoxon rank sum test). Box plot convention as in Figure S5. N indicates the number of units.*

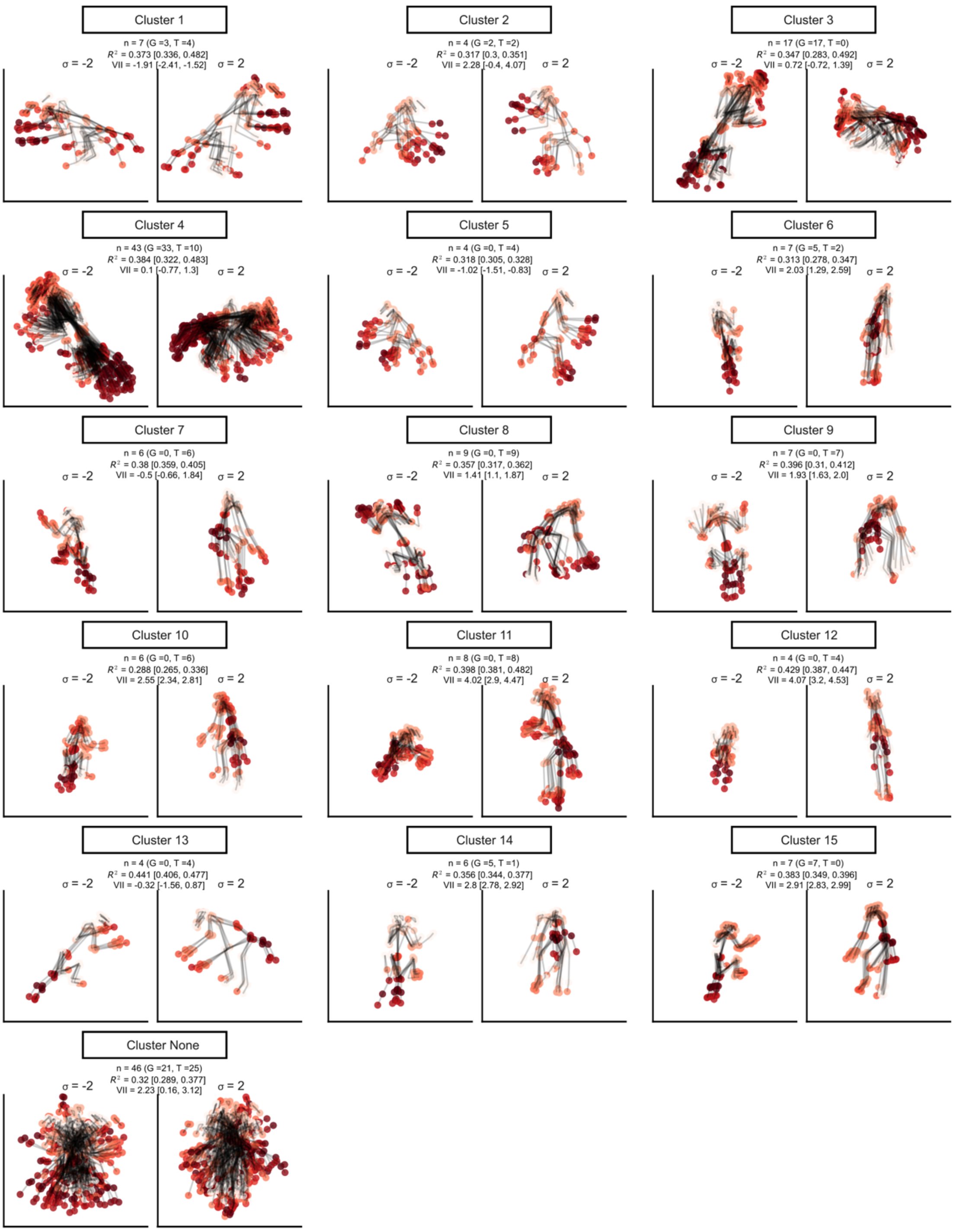

Figure S11. Clusters of units based on their preferred axes in the MSB region of both monkeys. *Each cluster includes all the poses within the cluster for the standard deviation values σ=-2 and σ=2 along the axis. For each cluster, n corresponds to the size of the cluster. As in Figures 3d and 3h, these poses were estimated using a 2D keypoint model, wherein the color of the keypoints represents their weight. Example units 258 and 625 of Figure 3 belong to clusters "None" and 4, respectively.*

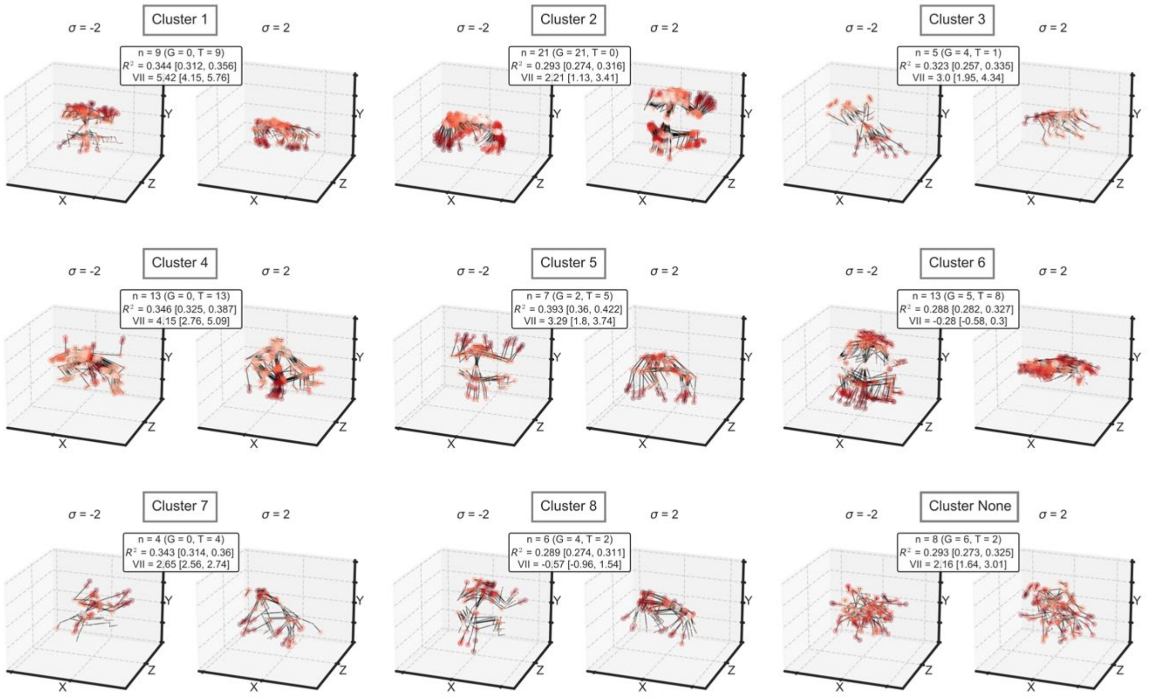

Figure S12. Clusters of units based on their preferred axes in the ASB region of both monkeys. *Each cluster includes all the poses within the cluster for the standard deviation values σ=-2 and σ=2 along the axis. For each cluster, statistics are shown where n corresponds to the size of the cluster. As in main Figure 3l, these poses were estimated using a 3D_VD keypoint model, with the color of the keypoints representing their weight. The example ASB unit of Figure 3 belongs to cluster 1.*

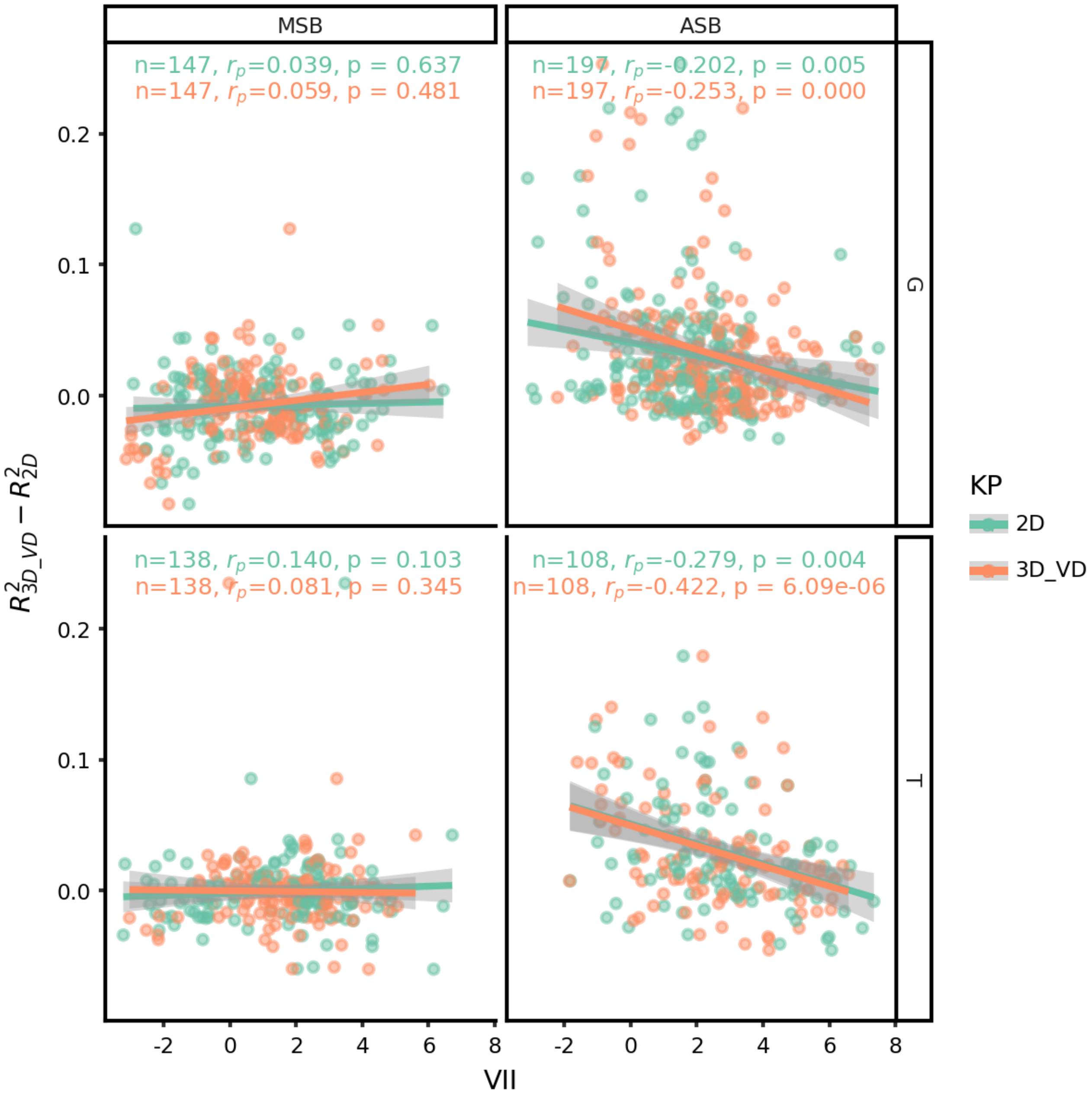

Figure S13. Scatterplots showing the relationship between the View Invariance Index (VII) and the difference in R^2 between the 3D_VD and 2D models. *Colors represent the model used to calculate the VII. Regression lines are plotted with the shaded region representing the 95% confidence interval. r_p corresponds to the partial correlation between VII and the difference in the coefficient of determination, with R^2 (of 2D or 3D_VD keypoint (KP) model, as indicated by colors) as control variable. All units were included. n indicates the number of units. ASB, MSB: regions; G, T: monkey subjects.*

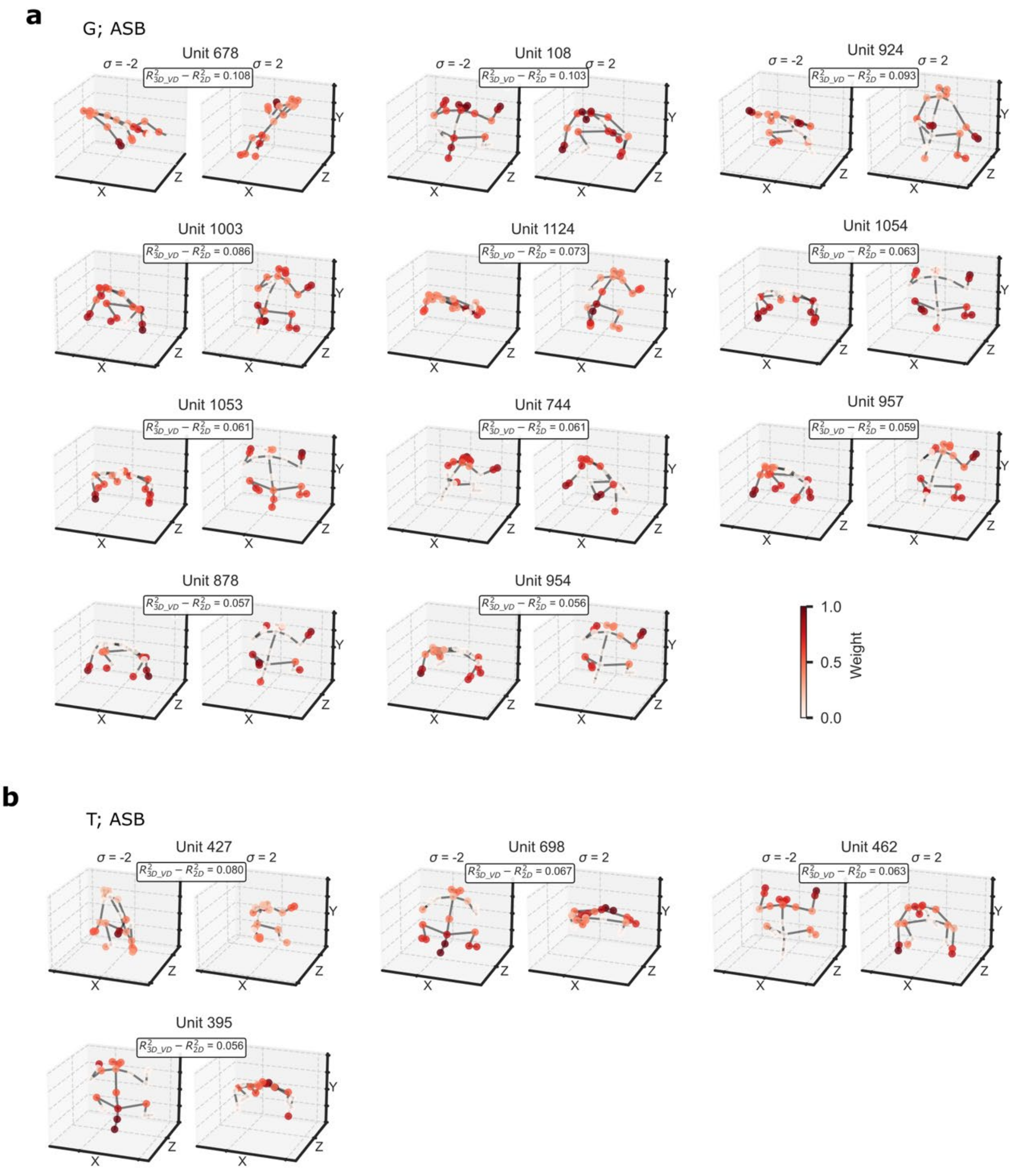

***Figure S14. Poses of units, not presented in main Figure 7, of ASB with an improved predictive performance for the 3D_VD model compared to the 2D model. a**, Poses of units in the ASB region of monkey G, for standard deviation values σ=-2 and σ=2 along the preferred axis, where the 3D_VD model provides a better fit compared to the 2D model. The difference in R^2 between the 3D_VD and 2D models is shown, with keypoint weights indicated by color. **b**, ASB units of monkey T. For each unit, R^2 for the 3D_VD model was larger than 0.25.*

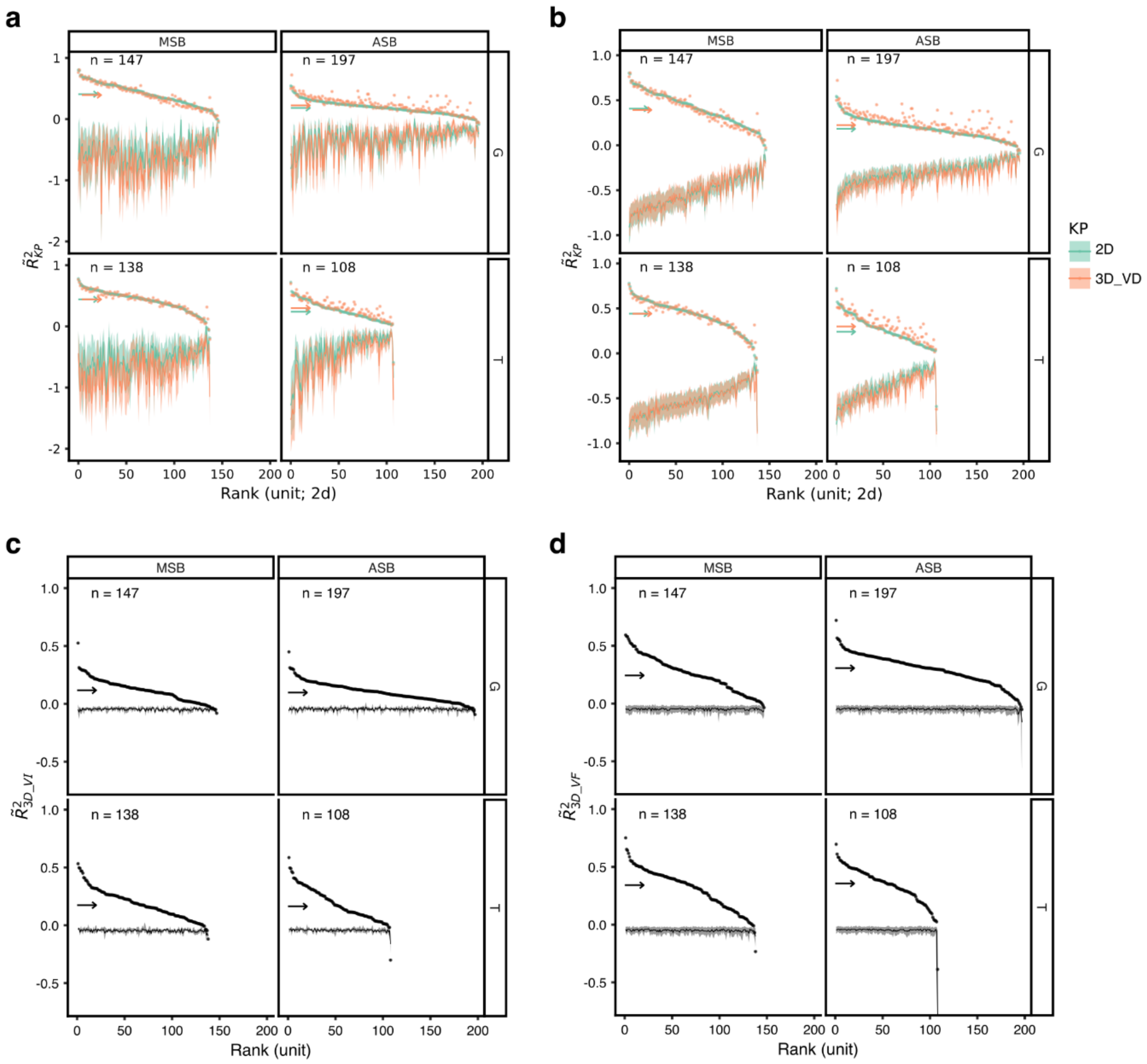

Figure S15. Ranked reliability-normalized coefficients of determination ($\tilde{R}^2_{KP}$). *$\tilde{R}^2_{KP}$ for all units in the 2D and 3D_VD keypoint models (as in Figure 2a), compared with the null distribution (range of percentiles 2.5 and 97.5) of $\tilde{R}^2_{KP}$ (shaded region), estimated using* ***a,*** *beta coefficient permutation and* ***b,*** *test label permutation approaches (see Methods for details).* ***c,*** *Ranked reliability-normalized coefficients of determination for all units fitted with the 3D_VI and* ***d,*** *with the 3D_VF model, compared with the null distribution estimated using permutation of both train and test stimulus labels (as in Figure 2a). Permutations were performed 100 times. The horizontal arrows indicate the median $\tilde{R}^2$. Data for all units of each monkey (G, T) and region (MSB, ASB). N indicates the number of units.*

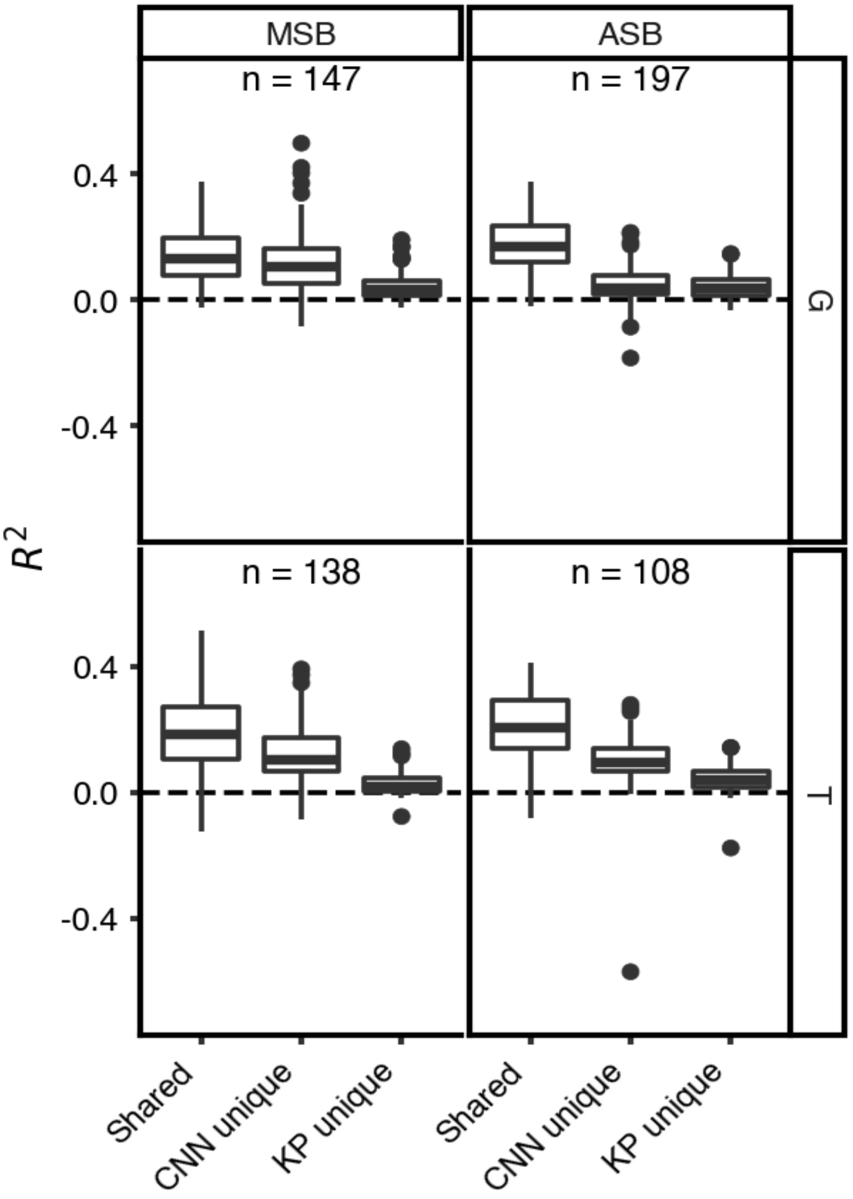

Figure S16. Variance partitioning analysis of AlexNet (CNN) and 3D_VF keypoint models. *The AlexNet activations were obtained from layer 6 (1st fully connected layer), which has been shown to have units with mirror-symmetric viewpoint tuning for objects and faces (Farzmahdi, A., Zarco, W., Freiwald, W.A., Kriegeskorte, N. & Golan, T. Emergence of brain-like mirror-symmetric viewpoint tuning in convolutional neural networks. Elife* ***13*** *(2024).). The results for AlexNet layer 6 are similar to those of Figure S9, showing shared variance between AlexNet and the 3D_VF model, in addition to unique explained variance for each model. All units were included, indicated by n. Box plot convention as in Figure S5.*

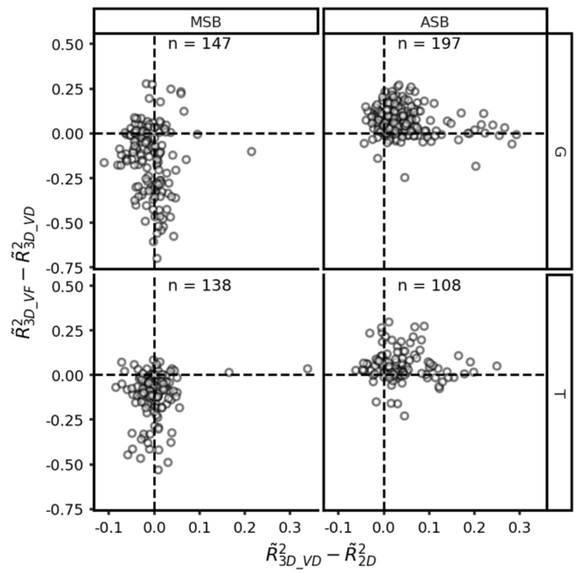

Figure S17. Scatterplots showing the relationship between $\widetilde{R}^2_{3D\_VD} - \widetilde{R}^2_{2D}$ and $\widetilde{R}^2_{3D\_VF} - \widetilde{R}^2_{3D\_VD}$ for each region and monkey. *Note the different scales of the x and y axes. N indicates the number of units.*

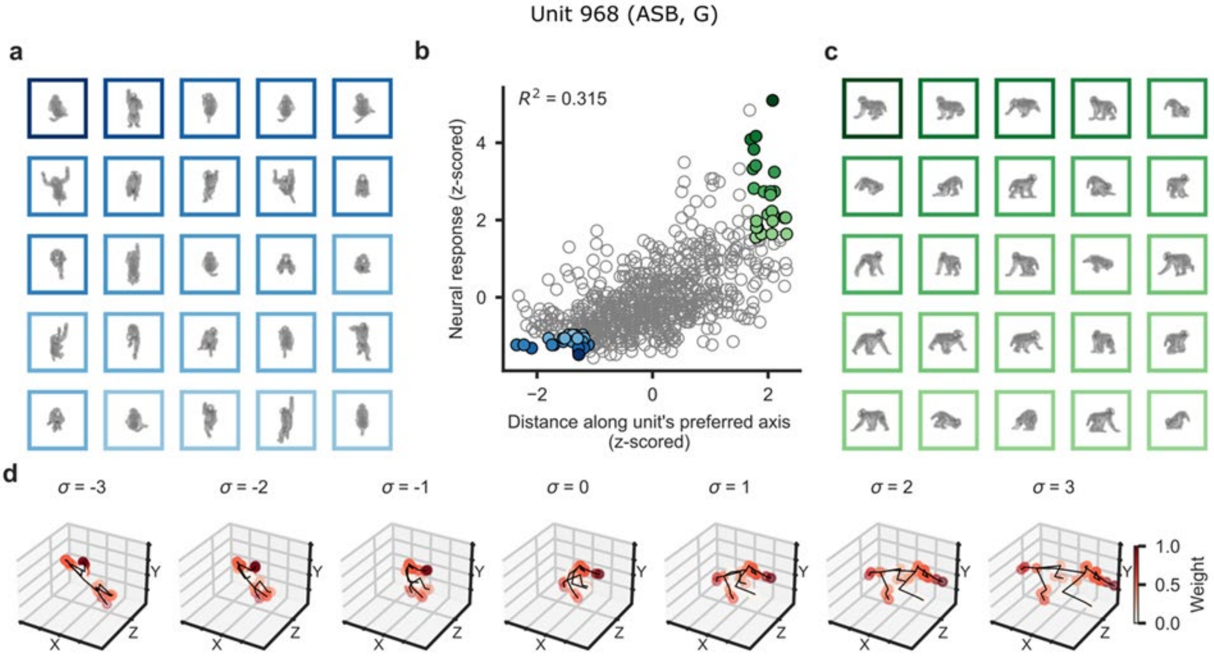

***Figure S18. The preferred axis of the unit with the highest difference in predictive performance between the 3D_VF and the 3D_VD model in the ASB region of G. a-d**, Data for unit 968. **b**, A scatter plot depicting the relationship between the unit's distance on its preferred axis (model response) and its actual response. **c**, Stimuli that elicited a high response, as shown in (**b**), are ordered in descending response strength (coded in shades of green; darker: stronger response). **a**, Stimuli that elicited a low response, as shown in (**b**), are ordered in ascending response strength (blue). **d**, Poses, estimated using the 2D keypoint model, corresponding to a range of standard deviations [σ=-3, σ=3] along the preferred axis. The color of a keypoint indicates its max-normalized weight, representing its contribution to the unit's selectivity. It was assigned a value of 0 when the observed weight of the keypoint was not significant (False Discovery Rate corrected). Conventions as in Figure 3 (main text).*

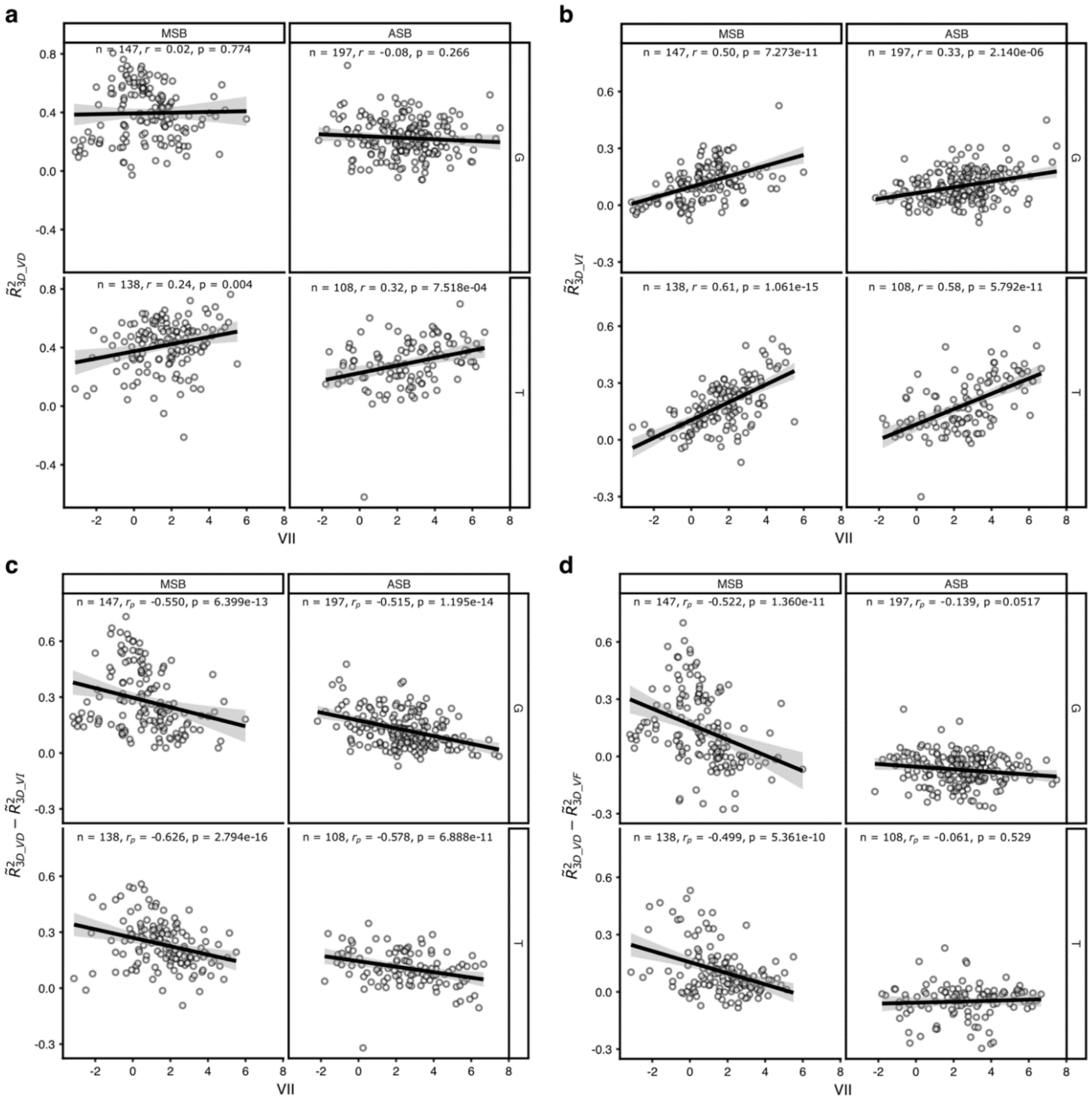

Figure S19. View Invariance Index (VII) and model performance. *Scatterplots showing the relationship between VII and **a**, $\tilde{R}^2_{3D\_VD}$, **b**, $\tilde{R}^2_{3D\_VI}$, **c**, the difference in $\tilde{R}^2$ between the 3D_VD and 3D_VI models, and **d**, the difference in $\tilde{R}^2$ between the 3D_VD and 3D_VF models. VII was computed using the 3D_VD model. A regression line is plotted for each condition, with the shaded region representing its 95% confidence interval. r_p (in **c** and **d**) corresponds to the partial correlation between VII and the difference in the coefficients of determination, with $\tilde{R}^2_{3D\_VD}$ as control variable. For VII and $\tilde{R}^2_{3D\_VI}$ (**b**), the partial correlations between these two variables, with $\tilde{R}^2_{3D\_VD}$ as control variable, were: MSB, G: $r_p = 0.56$; p = 3.24e-13; MSB, T: 0.62; p = 6.55e-16; ASB, G: 0.51; p = 1.56e-14; ASB, T: 0.57; p = 1.22e-10. The difference between the 3D_VD and 3D_VF model fits correlated significantly with the VII in MSB, with units showing a relatively worse 3D_VF predictive performance having a lower VII (**d**). No such correlation was present for ASB. Data of all units are plotted. N indicates the number of units.*

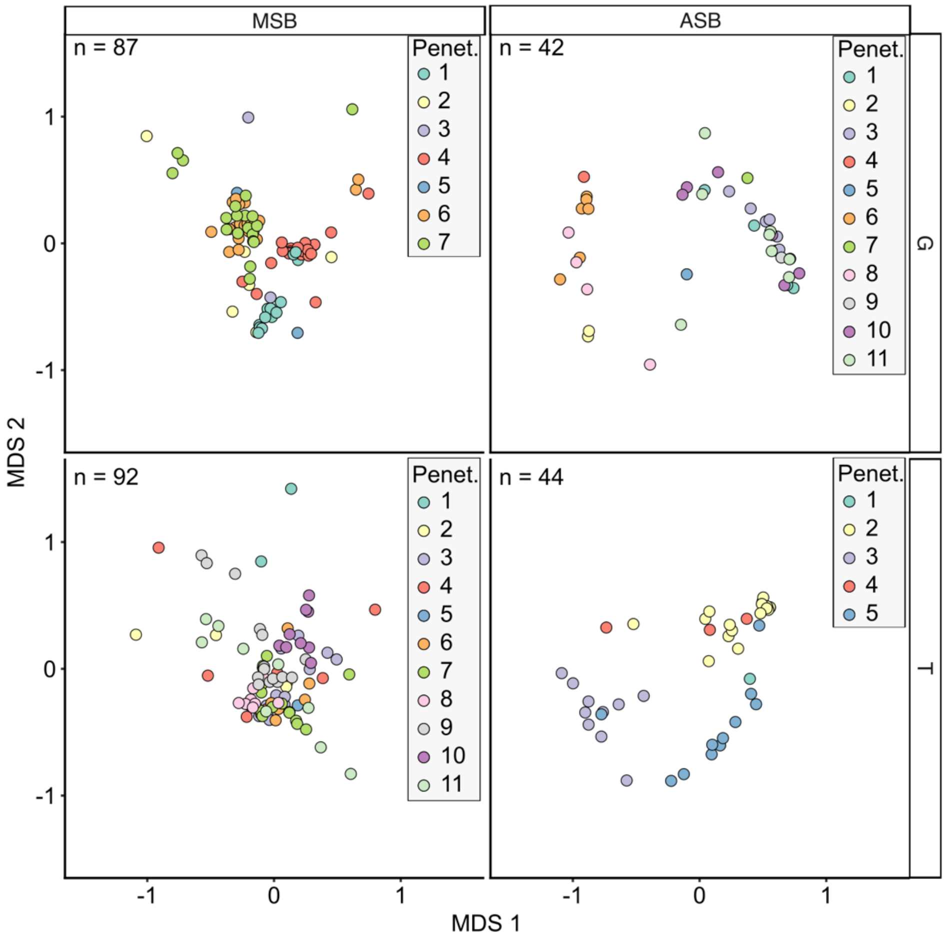

Figure S20. Anatomical clustering of preferred axes. *We computed for those units for which R^2 for the 3D_VD model was higher than 0.25 (the same criterium as for the plots of the axes in Figures 5-6), the pairwise cosine dissimilarity between the preferred axes (based on the 10 beta coefficients of the fitted model) and then applied multidimensional scaling to visualize the distances between those units in a two-dimensional space. Note that the 3D_VD model performed similarly to the 2D model for MSB units. We labeled the units according to the daily recording session (Penet.) they belonged to. There is a tendency for clustering of units from the same session in both regions and monkeys. The penetrations are estimated to be in a small volume of the STS in each region (range of grid positions on average (across monkeys and regions) 2 mm in the medial-lateral dimension and 1 mm in the anterior-posterior dimension), probes can bend along their trajectory, and penetrations were not orthogonal to the cortex because of the probe trajectory angles and curvature of the STS. Hence, an anatomical reconstruction of recording locations at columnar resolution is outside the scope of the present study. Nonetheless, the tendency for clustering of the preferred axes per session agrees with previous studies reporting clustering of stimulus preferences in IT (Fujita, I., Tanaka, K., Ito, M. & Cheng, K. Columns for visual features of objects in monkey inferotemporal cortex. Nature* ***360****, 343-346 (1992); Dubois, J., de Berker, A.O. & Tsao, D.Y. Single-unit recordings in the macaque face patch system reveal limitations of fMRI MVPA. J.Neurosci.* ***35****, 2791-2802 (2015). Verhoef, B.E., Vogels, R. & Janssen, P. Inferotemporal cortex subserves three-dimensional structure categorization. Neuron* ***73****, 171-182 (2012)). N indicates the number of units.*

monkey	region	model dim	VD	VF	VI
G	MSB	2D	0.407379	0.235372	
		3D	0.392706	0.243431	0.116733
	ASB	2D	0.182733	0.247509	
		3D	0.223607	0.306711	0.097264
T	MSB	2D	0.442057	0.345631	
		3D	0.441656	0.341770	0.174058
	ASB	2D	0.240174	0.304700	
		3D	0.298292	0.355809	0.163596

Table S1. Median reliability-normalized $\widetilde{R}^2_{KP}$ for the examined keypoint models for each monkey (G, T) and region (MSB, ASB). *Dim indicates whether 2D or 3D keypoint coordinates were employed. VD: 2D and 3D_VD model; VF: 2D_VF and 3D_VF model; VI: 3D_VI model.*

Supplementary text related to Figure S9. *The shared and unique variances were computed by comparing the variance explained by a concatenation of two models and the variance explained by each model (Methods). We used the "layer 5" activations of both networks since these produced a relatively high predictive performance for both models and regions (Figure S6c). The coefficients of determination were not normalized by reliability. Box plot convention as in Figure S5. N indicates the number of units.*

For MSB, the shared variance of the 2D/3D_VD models and the CNN models were higher than the unique variance for the CNNs and the latter was higher than the unique variance for the keypoint models. However, the keypoint models explained unique variance of the MSB responses, although it was numerically small. Overall, this result suggests that the keypoint models encode shape differences among the images, due to changes in pose and viewpoint, that are also captured by the CNNs. The unique variance explained by the CNNs, much smaller than the shared variance component, likely results from responses to texture and shading, which are absent in the purely shape-based keypoint models. The small unique variance component of the keypoint model might capture shape differences not encoded by the texture-biased CNNs. (The 3D_VF model produced a worse predictive performance than the 2D and 3D_VD models in MSB, and will not be discussed further).

For ASB, the amount of shared variance and the amount of unique variance of the CNNs was highly similar, in line with the relatively better predictive performances of the CNNs compared to the keypoint models, perhaps reflecting a relatively higher contribution of shading and/or texture cues to the selectivity in ASB compared to MSB. This needs confirmation in further studies. Note that this hypothesis aligns with the higher contribution of shape compared to "appearance" to face selectivity in the midSTS face patch ML while the opposite is the case in the anterior face patch AM (Chang, L. & Tsao, D.Y. The Code for Facial Identity in the Primate Brain. Cell ***169****, 1013-1028.e1014 (2017)). However, there was still (a small amount of) unique variance explained by the 3D_VD keypoint model, likely reflecting shape encoding not captured by the CNNs. The 3D_VF model shared a relatively large amount of variance with the CNNs (see also Figure S16), but the unique variances were less than the shared one for both CNNs and the 3D_VF model for ASB.*

In sum, the variance partitioning analysis suggests that CNNs capture most, but not all, of the variance explained by the keypoint models. However, this does not reduce the value of keypoint models, because the keypoint models have as advantage that they are interpretable and their preferred pose/view selectivity can be visualized, which is very difficult for the CNNs.
