# Supplementary material for: Keypoint-based modeling reveals fine-grained body pose tuning in superior temporal sulcus neurons
Source: Nat Commun. 2025 Jul 1;16:5796. doi: 10.1038/s41467-025-60945-5 (PMC12214754; doi:10.1038/s41467-025-60945-5)
Supplement: Supplementary file 2 — Reporting Summary [file 41467_2025_60945_MOESM2_ESM.pdf]

nature portfolio

Corresponding author(s): Rufin Vogels

Last updated by author(s): 14/05/25

Reporting Summary

Nature Portfolio wishes to improve the reproducibility of the work that we publish. This form provides structure for consistency and transparency in reporting. For further information on Nature Portfolio policies, see our Editorial Policies and the Editorial Policy Checklist.

Statistics

For all statistical analyses, confirm that the following items are present in the figure legend, table legend, main text, or Methods section.

n/a	Confirmed	
☐	☒	The exact sample size (*n*) for each experimental group/condition, given as a discrete number and unit of measurement
☐	☒	A statement on whether measurements were taken from distinct samples or whether the same sample was measured repeatedly
☐	☒	The statistical test(s) used AND whether they are one- or two-sided<br>*Only common tests should be described solely by name; describe more complex techniques in the Methods section.*
☐	☒	A description of all covariates tested
☐	☒	A description of any assumptions or corrections, such as tests of normality and adjustment for multiple comparisons
☐	☒	A full description of the statistical parameters including central tendency (e.g. means) or other basic estimates (e.g. regression coefficient) AND variation (e.g. standard deviation) or associated estimates of uncertainty (e.g. confidence intervals)
☐	☒	For null hypothesis testing, the test statistic (e.g. *F*, *t*, *r*) with confidence intervals, effect sizes, degrees of freedom and *P* value noted<br>*Give P values as exact values whenever suitable.*
☒	☐	For Bayesian analysis, information on the choice of priors and Markov chain Monte Carlo settings
☒	☐	For hierarchical and complex designs, identification of the appropriate level for tests and full reporting of outcomes
☐	☒	Estimates of effect sizes (e.g. Cohen's *d*, Pearson's *r*), indicating how they were calculated

Our web collection on statistics for biologists contains articles on many of the points above.

Software and code

Policy information about availability of computer code

Data collection	Recordings were performed using Open Ephys software and tasks and tests were run with custom software written in MicroPython and visualized on-line using NI LabVIEW.
Data analysis	Spike-sorting was performed with Plexon Offline Sorter 4.6.2. Data were analyzed using MATLAB R2017b, R2023b, and Python 3.9.7. Data and analysis software to obtain core results is available in Code Ocean, linked to the paper. Other code is available on request.

For manuscripts utilizing custom algorithms or software that are central to the research but not yet described in published literature, software must be made available to editors and reviewers. We strongly encourage code deposition in a community repository (e.g. GitHub). See the Nature Portfolio guidelines for submitting code & software for further information.

Data

Policy information about availability of data

All manuscripts must include a data availability statement. This statement should provide the following information, where applicable:

- Accession codes, unique identifiers, or web links for publicly available datasets
- A description of any restrictions on data availability
- For clinical datasets or third party data, please ensure that the statement adheres to our policy

Data and analysis software to obtain the core results are availabe in Code Ocean, linked to the paper. The data iand code is at https://codeocean.com/capsule/2215416/tree

The data and code from which the figures and statistics are derived will be available after acceptance of the paper.

Research involving human participants, their data, or biological material

Policy information about studies with human participants or human data. See also policy information about sex, gender (identity/presentation), and sexual orientation and race, ethnicity and racism.

Reporting on sex and gender	*Use the terms sex (biological attribute) and gender (shaped by social and cultural circumstances) carefully in order to avoid confusing both terms. Indicate if findings apply to only one sex or gender; describe whether sex and gender were considered in study design; whether sex and/or gender was determined based on self-reporting or assigned and methods used. Provide in the source data disaggregated sex and gender data, where this information has been collected, and if consent has been obtained for sharing of individual-level data; provide overall numbers in this Reporting Summary. Please state if this information has not been collected. Report sex- and gender-based analyses where performed, justify reasons for lack of sex- and gender-based analysis.*
Reporting on race, ethnicity, or other socially relevant groupings	*Please specify the socially constructed or socially relevant categorization variable(s) used in your manuscript and explain why they were used. Please note that such variables should not be used as proxies for other socially constructed/relevant variables (for example, race or ethnicity should not be used as a proxy for socioeconomic status). Provide clear definitions of the relevant terms used, how they were provided (by the participants/respondents, the researchers, or third parties), and the method(s) used to classify people into the different categories (e.g. self-report, census or administrative data, social media data, etc.) Please provide details about how you controlled for confounding variables in your analyses.*
Population characteristics	*Describe the covariate-relevant population characteristics of the human research participants (e.g. age, genotypic information, past and current diagnosis and treatment categories). If you filled out the behavioural & social sciences study design questions and have nothing to add here, write "See above."*
Recruitment	*Describe how participants were recruited. Outline any potential self-selection bias or other biases that may be present and how these are likely to impact results.*
Ethics oversight	*Identify the organization(s) that approved the study protocol.*

Note that full information on the approval of the study protocol must also be provided in the manuscript.

Field-specific reporting

Please select the one below that is the best fit for your research. If you are not sure, read the appropriate sections before making your selection.

☒ Life sciences ☐ Behavioural & social sciences ☐ Ecological, evolutionary & environmental sciences

For a reference copy of the document with all sections, see nature.com/documents/nr-reporting-summary-flat.pdf

Life sciences study design

All studies must disclose on these points even when the disclosure is negative.

Sample size	The sample size was determined based on previous electrophysiological recording studies in monkeys and are standards in the field.
Data exclusions	We excluded unresponsive and unreliable units and units that were not body-category selective according to our criteria as described in the paper.
Replication	We employed two subjects and the data are presented for each subject separately. Each subject is a replication of the other subject.
Randomization	Stimuli were presented randomly and recordings from the posterior and anterior patches were interleaved.
Blinding	No blinding during data collection was present. However, spike sorting was performed blindly in the sense that the spike soring was agnostic about the stimuli.

Reporting for specific materials, systems and methods

We require information from authors about some types of materials, experimental systems and methods used in many studies. Here, indicate whether each material, system or method listed is relevant to your study. If you are not sure if a list item applies to your research, read the appropriate section before selecting a response.

Materials & experimental systems

n/a	Involved in the study
☒	☐ Antibodies
☒	☐ Eukaryotic cell lines
☒	☐ Palaeontology and archaeology
☐	☒ Animals and other organisms
☒	☐ Clinical data
☒	☐ Dual use research of concern
☒	☐ Plants

Methods

n/a	Involved in the study
☒	☐ ChIP-seq
☒	☐ Flow cytometry
☐	☒ MRI-based neuroimaging

Animals and other research organisms

Policy information about studies involving animals; ARRIVE guidelines recommended for reporting animal research, and Sex and Gender in Research

Laboratory animals	2 male rhesus monkeys (Macaca mulatta), age: 7 years
Wild animals	n/a
Reporting on sex	sex was not part of the study design.
Field-collected samples	n/a
Ethics oversight	KULeuven animal ethical committee; protocol number P182/2019.

Note that full information on the approval of the study protocol must also be provided in the manuscript.

Plants

Seed stocks	*Report on the source of all seed stocks or other plant material used. If applicable, state the seed stock centre and catalogue number. If plant specimens were collected from the field, describe the collection location, date and sampling procedures.*
Novel plant genotypes	*Describe the methods by which all novel plant genotypes were produced. This includes those generated by transgenic approaches, gene editing, chemical/radiation-based mutagenesis and hybridization. For transgenic lines, describe the transformation method, the number of independent lines analyzed and the generation upon which experiments were performed. For gene-edited lines, describe the editor used, the endogenous sequence targeted for editing, the targeting guide RNA sequence (if applicable) and how the editor was applied.*
Authentication	*Describe any authentication procedures for each seed stock used or novel genotype generated. Describe any experiments used to assess the effect of a mutation and, where applicable, how potential secondary effects (e.g. second site T-DNA insertions, mosiacism, off-target gene editing) were examined.*

Magnetic resonance imaging

Experimental design

Design type	block design
Design specifications	The number of runs depended on the subject. Blocks of the six stimulus condition were repeated twice per run and the baseline fixation target -only condition was presented three times per run. The stimuli were presented back to back. The design was identical to that of Bognar et al , NeuroImage, 2023.
Behavioral performance measures	The monkeys were required to fixate for a juice reward. No other behavioral parameters were measured.

Acquisition

Imaging type(s)	functional
Field strength	3T
Sequence & imaging parameters	Functional MRI images were acquired using a custom-made 8-channel monkey coil, a saddle-shaped radial transmit-only surface coil , and a gradient-echo T2*-weighted echo-planar imaging sequence of 40 slices and flip angle of 90° (repetition time TR = 2000 ms, echo time TE = 18 ms, 1.25 mm isotropic voxel resolution). Slices were oriented transversally covering the whole brain. We obtained high-resolution anatomical MRI images for each monkey in a separate session under Ketamine/ Medetomidine anesthesia, using a single radial transmit–receive surface coil and a Magnetization-Prepared Rapid Acquisition with Gradient Echo (MPRAGE) sequence (TR = 2700 ms, TE = 3.5 ms, flip

angle = 9°, 208 slices, 0.4 mm isotropic voxel resolution). To increase the signal-to-noise ratio, we injected the contrast agent Monocrystalline Iron Oxide Nanoparticle (MION; Molday ION; 8–11 mg/kg) into the femoral/saphenous vein immediately before scanning.

Area of acquisition: whole brain

Diffusion MRI: ☐ Used ☒ Not used

Preprocessing

Preprocessing software: custom-software;

Normalization: Data were analyzed for each subject separately, using the anatomy of each subject.

Normalization template: Data were analyzed for each subject separately, using the anatomy of each subject.

Noise and artifact removal: A non-rigid, slice-by-slice realignment within runs and affine realignment between runs within a day was performed for motion-correction with jip-align (https://www.nmr.mgh.harvard.edu/?jbm/jip). Mean functional images were then non-rigidly co-registered to the T1 anatomical images of the same monkey in SPM12. Images were smoothed in SPM12 with an isotropic Gaussian kernel (FWHM: 1.5 mm).Subsequent data analysis was performed with SPM12. All valid runs were combined in a fixed-effects model for each subject separately in their native space. They were analyzed with a general linear model (GLM) with 7 regressors (6 stimulus conditions + fixation condition), plus 9 additional head-motion (6 covariates: translation and rotation in 3 dimensions) and eye-movement regressors (2 covariates for the horizontal and vertical eye position, and one for the pupil diameter) per run. Each condition was modeled using a convolution with a Gamma function (delta = 0, tau = 8 and exponent = 0.3), modeling the MION hemodynamic response function.

Volume censoring: SPM12

Statistical modeling & inference

Model type and settings: GLM

Effect(s) tested: univariate contrasts.

Specify type of analysis: ☒ Whole brain ☐ ROI-based ☐ Both

Statistic type for inference (See Eklund et al. 2016): voxel-wise t tests.

Correction: FWE correction

Models & analysis

n/a	Involved in the study
☒	☐ Functional and/or effective connectivity
☒	☐ Graph analysis
☒	☐ Multivariate modeling or predictive analysis
